# Treatment of lung disease in alpha-1 antitrypsin deficiency: a systematic review

**DOI:** 10.2147/COPD.S130440

**Published:** 2017-05-02

**Authors:** Ross G Edgar, Mitesh Patel, Susan Bayliss, Diana Crossley, Elizabeth Sapey, Alice M Turner

**Affiliations:** 1 Therapy Services, University Hospitals Birmingham NHS Foundation Trust, Birmingham, UK; 2 Institute of Inflammation and Ageing, University of Birmingham, Birmingham, UK; 3 Division of Primary Care, University of Nottingham, Nottingham, UK; 4 Institute of Applied Health Research, University of Birmingham, Birmingham, UK; 5 Department of Respiratory Medicine, University Hospitals Birmingham NHS Foundation Trust, Birmingham, UK; 6 Department of Respiratory Medicine, Heart of England NHS Foundation Trust, Birmingham, UK

**Keywords:** alpha-1 antitrypsin deficiency, treatment, emphysema, transplantation

## Abstract

**Background:**

Alpha-1 antitrypsin deficiency (AATD) is a rare genetic condition predisposing individuals to chronic obstructive pulmonary disease (COPD). The treatment is generally extrapolated from COPD unrelated to AATD; however, most COPD trials exclude AATD patients; thus, this study sought to systematically review AATD-specific literature to assist evidence-based patient management.

**Methods:**

Standard review methodology was used with meta-analysis and narrative synthesis (PROSPERO-CRD42015019354). Eligible studies were those of any treatment used in severe AATD. Randomized controlled trials (RCTs) were the primary focus; however, case series and uncontrolled studies were eligible. All studies had ≥10 participants receiving treatment or usual care, with baseline and follow-up data (>3 months). Risk of bias was assessed appropriately according to study methodology.

**Results:**

In all, 7,296 studies were retrieved from searches; 52 trials with 5,632 participants met the inclusion criteria, of which 26 studies involved alpha-1 antitrypsin augmentation and 17 concerned surgical treatments (largely transplantation). Studies were grouped into four management themes: COPD medical, COPD surgical, AATD specific, and other treatments. Computed tomography (CT) density, forced expiratory volume in 1 s, diffusing capacity of the lungs for carbon monoxide, health status, and exacerbation rates were frequently used as outcomes. Meta-analyses were only possible for RCTs of intravenous augmentation, which slowed progression of emphysema measured by CT density change, 0.79 g/L/year versus placebo (*P*=0.002), and associated with a small increase in exacerbations 0.29/year (*P*=0.02). Mortality following lung transplant was comparable between AATD- and non-AATD-related COPD. Surgical reduction of lung volume demonstrated inferior outcomes compared with non-AATD-related emphysema.

**Conclusion:**

Intravenous augmentation remains the only disease-specific therapy in AATD and there is evidence that this slows decline in emphysema determined by CT density. There is paucity of data around other treatments in AATD. Treatments for usual COPD may not be as efficacious in AATD, and further studies may be required for this disease group.

## Introduction

Alpha-1 antitrypsin deficiency (AATD) is an inherited condition that predisposes patients to chronic obstructive pulmonary disease (COPD)^1^ albeit with considerable variability of clinical phenotype.^2^ The spirometric diagnosis of AATD-related COPD and “usual” (unrelated to AATD) COPD is identical, but it manifests at a younger age. Not all subjects develop pulmonary disease and those who do vary in presentation and subsequent decline.^3^ The first subjects observed with low alpha-1 antitrypsin (AAT) levels were young smokers with basal panacinar emphysema,^4^ a finding which remains a typical AATD presentation,^5^,^6^ though bronchiectasis, neonatal jaundice, liver cirrhosis, and panniculitis may be seen.^6^–^9^

The most well-known polymorphisms (protease inhibitor [Pi] Z allele), present in the homozygous state in around 1/5,000 European Caucasians,^10^ arise from a point mutation in *SERPINA1* and result in a change to AAT structure, causing polymerization, accumulation in hepatocytes, and thus reduced circulating level of AAT.^11^ Homozygous “Z” patients have an AAT level of 1.3–7.7 μM, considerably less than the putative protective threshold of 11 μM typical of PiSZ patients.^12^ The primary function of AAT is protecting the lung from proteolytic enzymes, primarily neutrophil elastase (NE);^13^ in deficiency, uninhibited NE can therefore lead to lung damage via quantum proteolysis.^14^,^15^

Management of symptomatic lung disease is generally similar to usual COPD, including smoking cessation, inhalers, and pulmonary rehabilitation (PR).^16^,^17^ Infusion of plasma-derived AAT (augmentation therapy) to restore physiological levels is the only licensed disease-specific treatment and the only area studied by previous systematic reviews. Its use is variable worldwide, largely due to differing health systems, although controversy over efficacy exists.^18^,^19^ A review by Chapman et al included many different study designs, focused on forced expiratory volume in 1 s (FEV_1_) as an outcome measure, and concluded that augmentation slowed FEV_1_ decline relative to placebo;^18^ however, FEV_1_ has limitations, meta-analysis of varied study designs could have flaws, and major trials have been published since. Cochrane considered only randomized controlled trials (RCTs) and reviewed more outcomes (FEV_1_, diffusing capacity of the lungs for carbon monoxide [DLCO], computed tomography [CT] density, and quality of life [QoL]), concluding that augmentation was not beneficial, due to a lack of effect on lung function and QoL.^19^ However, the benefit of measuring lung density by quantitative CT scan analysis was that it relates to mortality in AATD,^20^,^21^ subsequently resulting in the review being criticized widely by specialists. As such, a new, more wide ranging review was indicated. In general, COPD meta-analyses demonstrating the impact of pharmacological and nonpharmacological interventions have been published,^22^,^23^ but most studies in usual COPD have excluded AATD patients, so the evidence may not be generalizable.

## Methods

The systematic review protocol is registered with PROSPERO (CRD42015019354). Standard systematic review methodology was used, aimed at minimizing bias, with reference to the Cochrane Handbook for Systematic Reviews of Interventions.^24^

### Search strategy

The following databases were searched by using no date or language restrictions (Supplementary materials): MEDLINE, MEDLINE In Process and EMBASE (via Ovid), Cochrane Library (Wiley) CENTRAL, CDSR, HTA, EED, and DARE. In addition, Conference Proceedings Citation Index via Web of Science and British Library’s ZETOC were searched for conference proceedings and abstracts. ClinicalTrials.gov and World Health Organization International Clinical Trials Registry Platform were searched for ongoing trials. References of included studies and reviews were checked.

### Study selection criteria

**Figure 1:**
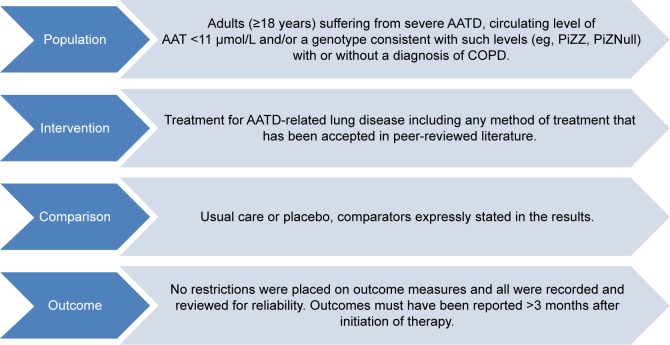
PICO chart detailing inclusion in systematic review. **Note:** PICO assessed are shown. **Abbreviations:** AATD, alpha-1 antitrypsin deficiency; AAT, alpha-1 antitrypsin; Pi, protease inhibitor; COPD, chronic obstructive pulmonary disease; PICO, population, interventions, comparators, and outcomes.

Figure 1 shows the selection criteria. Systematic reviews and primary study designs assessing treatment were eligible provided they included data on ≥10 participants with AATD. Nonsystematic reviews and preclinical studies were excluded. Studies comprising mixed populations were permitted if AATD data were available separately. Any intervention for AATD-related lung disease was eligible for inclusion. The only restriction placed on outcome measures was of reporting >3 months after initiation of therapy, thus limiting to the most clinically relevant studies for this chronic disease. Studies which, for instance, measured solely AAT concentration before and after augmentation dosing were not included, and this criterion ensured such studies were not selected.

### Data extraction and assessment of bias

**Figure 2:**
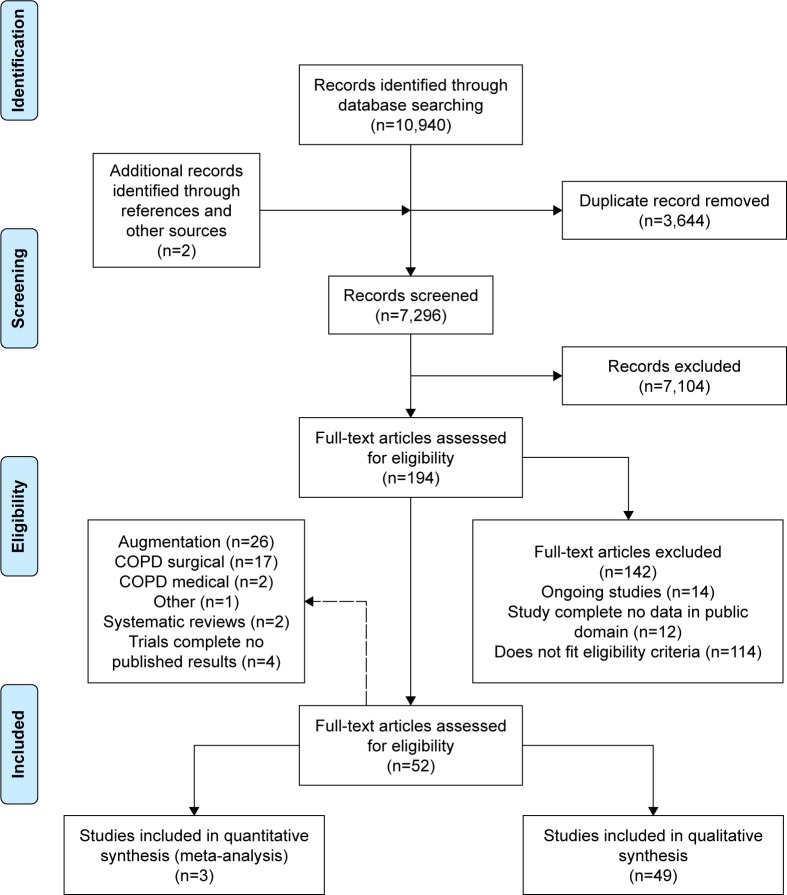
PRISMA flow diagram. **Notes:** Adapted from Moher et al.^25^ Articles were excluded where there were insufficient data in the public domain for the study to be assessed against the inclusion criteria including data from ClinicalTrials.gov. **Abbreviation:** COPD, chronic obstructive pulmonary disease.

Initial search yields (titles and abstracts) were screened for relevance by two reviewers independently, resolving any disagreement by discussion. Relevant articles were obtained and assessed against the full selection criteria, with translation of non-English language articles if required. Full-text articles were dually reviewed as before, with data extraction and bias assessment completed by one reviewer and checked by another. Missing data were requested from study authors if necessary (Supplementary materials). Reasons for excluding studies were documented and summarized in the PRISMA flow diagram (Figure 2).^25^

### Evidence synthesis

Most data were suitable only for narrative synthesis, due to heterogeneity of study design and outcomes. Studies were divided into subgroups to reflect four “themes”: AATD-specific, surgical, and medical treatments used in usual COPD, and other treatments. Data from three RCTs of AAT augmentation were meta-analyzed, the study design being sufficiently similar to allow this,^26^–^28^ including changes in lung density (measured by quantitative CT scan at least annually), FEV_1_ % predicted of normal, DLCO, QoL (St Georges Respiratory Questionnaire [SGRQ]), and annualized exacerbation rate (measured at least 6 monthly). Assessment of clinical and methodological heterogeneity was used to determine a fixed or random effect model; fixed effects using mean difference was appropriate in all but DLCO where a standardized mean difference was used. Differences between baseline and follow-up were synthesized and annualized; *I* 2 and τ^2^ statistics are reported where appropriate.

## Results

Following the removal of duplicates, 7,296 records were reviewed with 52 trials, comprising 5,632 participants’ data included in the final analysis (Figure 2). The two systematic reviews identified were briefly described in the “Introduction” section and justified this new review; hence, they will not be discussed further. The complete study characteristics of the remaining studies are shown in the Supplementary materials.

### AATD-specific treatment: augmentation

There were 26 eligible studies using a number of different product brands including Prolastin and Prolastin-C, Aralast, Zemaira, Trypsone, Respitin, and Glassia. Dosing regimes ranged from weekly to monthly with most using 60 mg/kg/week.

### RCTs

Three international multisite RCTs were identified,^26^–^28^ including a total of 320 participants (58^26^–180^28^ per study), conducted over 2–3 years, with optional subsequent open-label elements in two studies.^27^,^28^ All studies used CT density change as an outcome, two utilizing it as an experimental measure^26^,^27^ and one powering for CT density as the primary outcome (RAPID trial^28^). Other outcome measures included QoL, spirometry, and gas transfer and COPD exacerbation rates. Two studies used a standard dose (60 mg/kg body weight) intravenous infusion weekly,^27^,^28^ while the third used 250 mg/kg infused every 4 weeks.^26^

**Figure 3:**
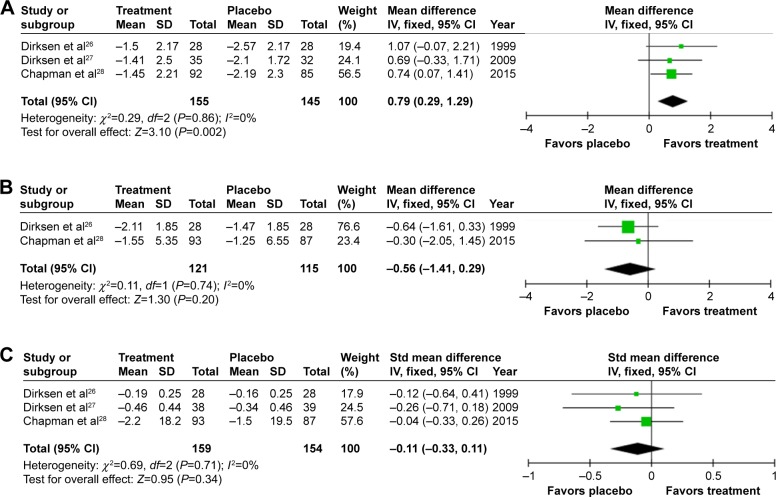
Forest plots of the objective results from meta-analysis of augmentation trials. **Notes:** (**A**) Mean annual change in lung density. (**B**) Mean FEV_1_ % predicted. (**C**) Standardized mean difference in DLCO. Differences in units used for DLCO (mmol/min/kPa and mL/mmHg/min) and the use of percentage change from baseline in RAPID, but annual change in the other studies required the use of a standardized mean difference plot. **Abbreviations:** FEV_1_, forced expiratory volume in one second (L); DLCO, diffusing capacity of the lungs for carbon monoxide; mmol/min/kPa, millimole per minute per kilopascal; mL/mmHg/min, milliliter per millimeter of mercury per minute; SD, standard deviation; CI, confidence interval; *df*, degrees of freedom; Std, standard; IV, inverse variance.

**Figure 4:**
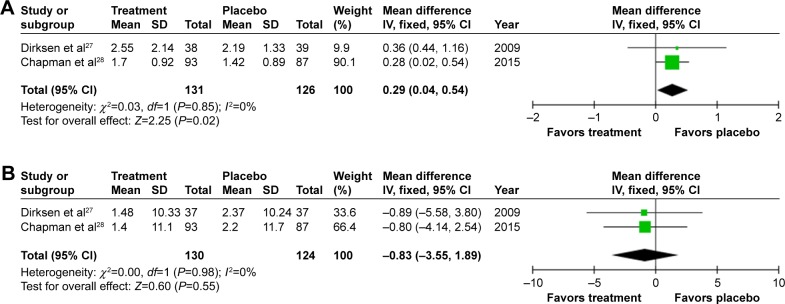
Forest plots for patient-reported outcomes. **Notes:** (**A**) Annual patient-reported exacerbation episodes. (**B**) Health status: SGRQ, measures recorded as change from baseline. **Abbreviations:** SGRQ, St Georges Respiratory Questionnaire; SD, standard deviation; CI, confidence interval; *df*, degrees of freedom; IV, inverse variance.

Mortality data were only reported by RAPID^28^ with one death on treatment and three on placebo. Adverse events (AEs; treatment related or not) were similar in the two studies that reported them;^27^,^28^ one did not report AEs.^26^ The remaining outcomes were possible to meta-analyze and are summarized in Figures 3 and 4. Heterogenicity was not detected (all *I*^2^=0%). In all studies, volume-corrected CT scans at total lung capacity were performed, this being the preferred and most validated scanning method,^27^,^29^–^31^ though three scanning methods were reported in RAPID. Lung density was analyzed by regression in the study by Dirksen et al^26^ and RAPID^28^ with four analysis methods in EXACTLE.^27^ EXACTLE’s “*method 1*”^27^ was used in this meta-analysis as it utilizes the same regression technique as other studies; sensitivity analyses using other EXACTLE methods did not alter the outcome or significance. Annual deterioration in lung density was less on augmentation; difference 0.79 g/L/year (95% confidence interval [CI] 0.29–1.29; *P*=0.002, Figure 3), demonstrating a slower rate of emphysema progression. There was no significant difference in annual FEV_1_% predicted decline on treatment (0.56% predicted/year [1.14–0.29; *P*=0.20]). A small nonsignificant difference of −0.11 (−0.33–0.11; *P*=0.34) in DLCO was observed.

Annual exacerbation rate was not reported in the earliest study,^26^ but there was a small, statistically significant increase in annual exacerbations on treatment (0.29/year [0.02–0.54; *P*=0.02], Figure 3) when meta-analyzing the other studies.^27^,^28^ Small and nonsignificant changes in health status were observed in both groups,^27^,^28^ demonstrating greater worsening in SGRQ on placebo 0.83 (−3.55 to 1.89; *P*=0.55, Figure 4).

### Observational controlled studies

There were six eligible controlled observational studies, comprising 2,610 participants. AEs and reasons for starting/stopping therapy were reported by one registry; severe events occurred at a rate of 9.5% (69/720 infusions).^32^

The largest observational study analyzed data from 1,129 patients in the US AATD registry split into three groups “always receiving” (n=390), “partly receiving” (n=357), or “never receiving” (n=382) augmentation.^33^ Dosing was not standardized with only 51.3% being dosed weekly throughout the study. A survival analysis was conducted, but excluded 81 subjects (55 deaths) due to missing data, such that results could have been biased. Overall mortality was 18.1% (n=204); it was significantly higher for subjects who never received augmentation therapy (as opposed to sometimes or always) when FEV_1_<50% predicted (*P*<0.001). Mortality rates were low for other subjects and did not differ between augmentation therapy groups. FEV_1_ decline was calculated using a slope equation in 927 patients with n=202 excluded due to insufficient data; patients receiving augmentation with mean FEV_1_ values of 35%–49% predicted had a slower rate of FEV_1_ decline (−73.7±6.8 vs −93.2±8.9; *P*=0.03), though this was not seen in the whole group.

Three other studies investigated the effect of AAT augmentation on FEV_1_ decline.^34^–^36^ Seersholm et al^34^ undertook a nonrandomized surveillance study in two cohorts. A statistically significant difference in FEV_1_ annual decline was observed (−53 [48–58] vs −75 [63–87] mL/year in treatment vs placebo; *P*=0.02).^34^ The other two studies concurred with this result. Wencker et al conducted a pre–post study of augmentation, using inclusion criteria of ≥2 lung function measurements prior to augmentation and two following commencement of therapy within a minimum period of 12 months.^36^ FEV_1_ declined significantly slower (−34.3±29.7 vs 49.2±60.8 mL/year, *P*=0.019) after starting augmentation. Tonelli et al compared 124 augmented PiZZ patients to 40 nonaugmented patients who had a median of two spirometry measurements over a mean follow-up of 41.7±2.6 months.^35^ Again, FEV_1_ decline was worse in untreated patients (+10.61±21.4 vs −36.96±12.1 mL/year; *P*=0.05). All three studies stratified patients to groups by their FEV_1_ at presentation – FEV_1_<30%, 30%–65%, and >65% predicted.^34^–^36^ Patients with FEV_1_<30% were consistently observed not to benefit from augmentation in terms of FEV_1_ decline. Two of the three studies showed those with an FEV_1_>65% to have statistically significant reductions in FEV_1_ decline when on augmentation (−122.5±108.4 vs −48.9±54.9 mL/year; *P*=0.045 and −108.7±17.3 vs −29.2±15.29 mL/year; *P*=0.0006).^35^,^36^ Treatment effect was restricted to patients with FEV_1_>30% and <65% (−62 [57–67] vs −83 [70–96] mL/year; *P*=0.04) in one study.^34^ When FEV_1_ at commencement of therapy was used to group patients, a statistically significant decrease of rate of decline during treatment was seen if FEV_1_<30% (53.4±45.3 to 22.1±16.0 mL/year; *P*<0.0001).^36^ Subgrouping the FEV_1_>65% group demonstrated marked benefit in those deemed rapid decliners (FEV_1_ decline pre- vs postaugmentation −255.7±70.4 vs 52.7±61.3 mL/year; *P*=0.0016).

The most recent study by Barros-Tizon et al^37^ was a retrospective medical records review of 127 participants evaluating the effect of augmentation on exacerbation rate. Seventy-five patients had ≥1 exacerbation during the 18 months follow-up required prior to commencement of augmentation. Dosing regimens were not standardized with an average dose of 60.7±3.8 mg/kg/week (Table S1) different from other studies,^33^ and many patients had missing data. Fewer exacerbations were seen (1.2±1.6 vs 1.0±2.2 pre- vs posttreatment; *P*<0.01), an effect more marked in those exacerbating previously (2.0±1.6 vs 1.4±2.7; *P*<0.01).

### Controlled studies of differing dose and/or drug manufacturer

Five RCTs^38^–^42^ with 176 participants investigated differences between dosing regimens of a product^38^ or made comparisons between different products.^39^–^42^ Campos et al^38^ used a double-blind crossover study of 60 versus 120 mg/kg/week of Prolastin-C to investigate safety and pharmacokinetics in 30 patients. They reported no increase in AEs, a higher trough serum AAT concentration, and that the higher dose was tolerated well by patients. Four double-blind RCTs^39^–^42^ completed investigating pharmacokinetic equivalence between weekly dosing of the new investigational product (Respitin,^42^ Zemaira,^41^ Prolastin-C,^39^ and Glassia^40^) and control (60 mg/kg/week Prolastin). All the studies had an optional open-label continuation study for safety data collection (ranging from 8 weeks^39^ to 2 years^41^) and reported no significant difference in AEs between the investigational product and control. Pharmacokinetic equivalence was reported in all studies with none demonstrating superiority over Prolastin. Stoller et al,^42^ and Sandhaus et al^40^ reported no significant differences in lung function parameters between the investigation groups or over the duration of the studies. One death was reported (respiratory arrest related to COPD) in the study by Stocks et al,^41^ which was in the control arm and considered unrelated to study medication.

### Observational uncontrolled studies

Twelve uncontrolled observational studies using intravenous AAT augmentation in 2,526 patients were included, which assessed safety and tolerability,^43^–^45^ pharmacokinetics,^46^–^48^ pulmonary neutrophilic inflammation,^49^ longitudinal change in lung function,^50^–^52^ and clinical characteristics or recipients.^53^,^54^ AAT augmentation was reported as safe and well tolerated (one study drug-related AE out of 555 doses)^45^ with 26 AEs probably/possibly related to the study drug,^43^,^45^,^47^ largely comprising symptoms commonly observed in infusions of other protein-based products.^52^ Other adverse reactions reported^43^,^49^ were one widespread skin reaction,^49^ headache and increased shortness of breath, and hospital treatment for fever, hypotension, and hypoxemia.^43^ In all, 61 of 2,526 patients died with none attributed to AAT augmentation.^46^,^51^,^52^ Augmentation was again well tolerated and deemed safe in a small study of 60 mg/kg/week versus 240 mg/kg/month.^50^

Biochemical efficacy at the higher dose given monthly did not demonstrate a protective AAT level in 3 of 16 patients.^50^ A second study investigating the pharmacokinetics of an alternative dose (Prolastin, 120 mg/kg every 2 weeks) was completed in 23 participants;^46^ none of the patients maintained target serum AAT levels (>80 mg/dL) and only two maintained serum AAT levels >70 mg/dL from days 7 to 14 suggesting insufficient dosing. Wewers et al^47^ included 21 patients infused weekly for up to 6 months and demonstrated partial correction of biochemical abnormalities in serum (AAT trough level 126±1 vs 30±1 mg/dL) and pulmonary epithelial lining fluid (AAT 0.46±0.16 vs 1.89±0.17 μM). Similarly, effective concentrations of AAT were reported by two short-term studies^43^,^44^ and one 3-year study.^51^ A study investigating 18-fluorodeoxyglucose (18FDG) positron emission tomography–CT as a novel noninvasive biomarker^49^ had a subgroup of 10 patients with severe AATD. The study demonstrated that 12 weekly infusions of AAT augmentation therapy had no effect on circulating neutrophil 18FDG uptake and activity in the lung.

Pulmonary function was reported in five studies with no significant changes in rates of decline or spirometry being observed.^46^,^47^,^50^–^52^ An observational registry study where longitudinal FEV_1_ follow-up was available in 287 patients over 37.8±18.9 months showed significant differences in decline in FEV_1_<30% predicted and FEV_1_ 30%–65% groups (−35.6±21.3 vs −64.0±26.4 mL; *P*=0.0008).^52^

AlphaNet in USA is a not-for-profit health management company, which undertook a prospective study involving 922 members on augmentation, using monthly telephone interviews to collect data including exacerbation history and QoL (SGRQ) at baseline, months 6, and 12.^53^ Totally, 2,268 exacerbations (mean 2.45±1.3/subject/year) were observed, mostly graded to be of moderate severity. SGRQ did not change by the minimal clinically important difference (MCID) during the 12-month follow-up in any age group, though differences were seen according to age. The same cohort was used^54^ to describe exacerbations in more detail. Mean duration was 17.4±11.4 days by symptom-based classification, with a trend for increasing frequency and duration as disease severity increased; Global initiative for chronic Obstructive Lung Disease (GOLD) stage I and II patients had shorter, fewer exacerbations than GOLD stage III and IV (both *P*<0.05). Annual hospitalization for exacerbations was decreased in 4/14 patients in one study where study entry annual rates were used to assess augmentation; however, the study was insufficiently powered to establish the effectiveness in modifying disease.^43^

### COPD medical management

There were only two eligible studies in AATD using traditional COPD medical management strategies.^17^,^55^ The first reported good uptake and efficacy of influenza vaccination in the AlphaNet cohort (n=939),^55^ with 766 (81.6%) patients receiving vaccination, who then had fewer unscheduled outpatient department visits and critical care admissions (*P*=0.04 and 0.01, respectively). No statistically significant differences in exacerbation frequency or respiratory outcomes were observed. Another observational study by AlphaNet investigated the use of a multimodal self-management program with a combination of directed patient self-education, organized supervision, and health care provider education (Alpha-1 Disease Management and Prevention Program) in 1,028 participants.^17^ This 2-year study used data from the first “observational” year as comparator to a second year when the management plan was delivered. A total of 905 participants completed the 2 years demonstrating low attrition. There was strong evidence to support improved compliance in some medications (long-acting beta agonist [LABA] *P*<0.001, theophylline *P*=0.01, and systemic steroids *P*=0.02) and supplementary oxygen (*P*<0.01) along with reductions in annual exacerbation frequency and duration (*P*<0.001 and *P*=0.04, respectively). There were no significant changes to health status.

### COPD surgical management

#### Lung volume reduction (LVR)

Six studies investigated the use of Lung Volume Reduction Surgery (LVRS) in AATD. Five studies (n=71 patients) used an open surgical technique,^56^–^60^ and all demonstrated improvements in either physiological measurements or dyspnea. Benefits were inferior and shorter in duration than usual COPD patients in all studies. One small RCT randomized participants to LVRS (n=10) or medical treatment (n=6); higher 2-year mortality (20% vs 0%) occurred in the surgical group, albeit alongside improvements in SGRQ.^59^ There was one published study using endobronchial valves, which demonstrated their safety in AATD patients with significant benefits in mean FEV_1_ at 6 months, 1, and 2 years (*P*=0.0022, 0.0067, and 0.033, respectively).^61^ The generalizability of this study is not evident as this cohort included strict inclusion criteria including severe heterogeneous emphysema demonstrated by CT scan and scintigraphy, residual volume ≥140%, FEV_1_ 15%–45%, and optimal lobe selection. This resulted in fewer than half of the referrals meeting these criteria.^61^

#### Lung transplantation

Eleven studies over 24 years reported 2,146 lung transplants in AATD patients.^62^–^72^ Two studies investigated survival after transplantation compared with nontransplantation management; Tanash et al^71^ observed a survival benefit from transplant (11 [9–14] vs 5 [4–6] years; *P*=0.006). No significant difference was observed in survival (10.1 vs 8.4 years; *P*=0.954); however, improvements in total SGRQ (and all domains) were seen at 1 year (*P*<0.01).^72^ Six studies^64^–^69^ using retrospective reviews of lung transplant registries (all cause, not specific to AATD) contained sufficient separately reported data on mortality. Two demonstrated superior 10-year survival in AATD patients compared to usual COPD (*P*=0.04 and *P*<0.0001, respectively),^67^,^68^ but both noted that COPD recipients were often older with greater comorbidity. Conversely, Breen et al observed higher patient survival at 2 years in usual COPD (75.4%±4.4% vs 64.4%±5.4% in AATD); however, insufficient power precluded statistical analysis.^64^ All other studies showed no difference in long-term survival between AATD- and non-AATD-related COPD.^62^,^65^,^66^,^69^,^70^

There were some studies that reported other posttransplant outcomes. A subgroup analysis of patients receiving bilateral lung transplants demonstrated faster posttransplant FEV_1_ decline in AATD patients (*P*<0.002).^62^ Analysis of gastrointestinal complications posttransplant surgery showed that having AATD significantly increased the risk of requiring early postoperative laparotomy (odds ratio 5.74, 95% CI 2.15–15.35), which increased all-cause mortality by 62% (*P*>0.05).^63^

### Other management

A double-blind, parallel-group RCT of Palovarotene to reduce inflammation and promote structural repair in the lungs failed to demonstrate significant differences in change from baseline for CT density, FEV_1_, or gas transfer measures and exacerbation.^73^

### Unreported trials

A study of an inhaled AAT augmentation therapy (NCT01217671) is listed as completed, but limited data are currently available.^74^,^75^ One registered study (NCT00242385)^76^ compared two augmentation products in a double-blind, crossover RCT. Only one outcome (AEs) was eligible for inclusion as follow-up for all other outcomes were limited to 35 days; similar safety profile and no serious AEs were seen, but no statistics were available. A second study compared two Prolastin-C levels at 60 and 120 mg/kg to assess safety and pharmacokinetics (NCT01213043).^77^ No statistical analysis was available but 30 participants completed the study with similar treatment-emergent AEs and safety profile to other studies; no serious AEs occurred. There is currently one study (NCT01983241)^78^ investigating 60 and 120 mg/kg weekly administrations of Prolastin-C against placebo to determine safety and efficacy using change from baseline in CT lung density as the primary outcome.^79^

### Risk of bias

**Figure 5:**
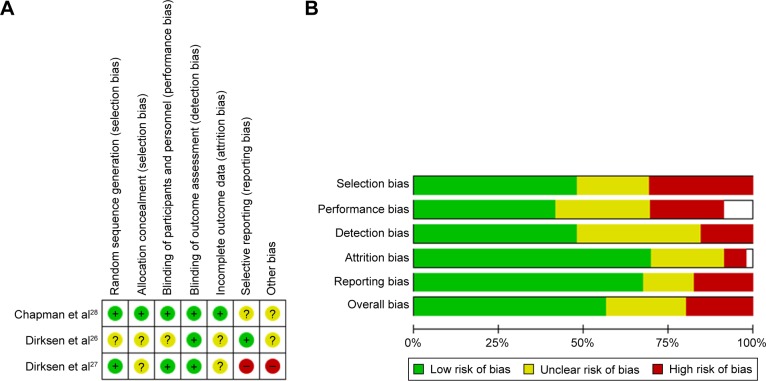
Risk of bias in included studies. **Notes:** (**A**) Risk of bias in augmentation RCTs. (**B**) In view of the large number of studies and the differing scales for assessment of risk based on the study design, a summary of the relative risk of bias is shown across all included studies, other than the RCTs of augmentation. This represents review authors’ judgments about each risk of bias item presented as the mean percentage across all included studies. Individual bias assessments are available in the Supplementary materials. **Abbreviation:** RCT, randomized controlled trial.

Risk of bias for the meta-analyzed RCTs is summarized in Figure 5A. Risk of bias in RAPID^28^ was low; however, in the two earlier augmentation studies, this was more difficult to assess and generally unclear due to poor reporting, specifically of details such as allocation concealment. The other six RCTs demonstrated low or unclear bias, mostly due to lack of detail in the manuscripts, commonly around blinding. Figure 5B summarizes the risks in the remaining studies. The mixture of RCTs and cohort and observational studies (both prospective and retrospective) led to an overall moderate risk of bias especially in the nonblinded studies. In the controlled or quasi-controlled studies, there was often insufficient reporting of the blinding process and allocation concealment, except in more recent studies, and a trend toward selective outcome reporting.^18^,^36^,^38^ Within the uncontrolled retrospective studies, selection bias was the main issue, with patients being taken from self-selecting groups or registries. Transplant studies from registries suffered from high attrition rates, which may have introduced survivor bias.^64^–^69^

## Discussion

The available evidence for the management of AATD was largely centered on augmentation, which seemed to benefit emphysema, but with significant deficits in the evidence base for outcomes more typically assessed in COPD studies, such as exacerbation frequency and QoL. Although it is not unreasonable to think that strategies used to treat COPD unrelated to AATD could be extrapolated to AATD patients, there was almost no evidence proving this.

### Augmentation

Augmentation was demonstrated to be safe and well tolerated in numerous studies using different products. Investigations into dosing regimes demonstrated few benefits from >60 mg/kg/week^38^ (the “standard” dose), and pharmacokinetic equivalence was observed between different manufacturers’ products.^40^,^41^

The meta-analysis confirmed that augmentation therapy is able to slow down the progression of severity of emphysema when measured by CT density change compared with placebo (*P*=0.002). By combining data from up to 320 patients included in three RCTs, it adds to an increasing body of literature demonstrating the usefulness of CT densitometry as a surrogate measure of emphysema and outcome in both AATD and usual COPD.^20^,^80^,^81^ However, the MCID has not yet been established for change in CT density, which would be helpful for interpretation. Meta-analysis also revealed a small but significant increase in annual exacerbation rate on augmentation. This is somewhat counterintuitive and contradicted by studies included in narrative synthesis, which either showed a benefit or no significant difference in exacerbation rate or severity.^17^,^37^,^54^ Increased contact with health care professionals, simply due to attending for infusions, could lead to increased reporting of exacerbations, but this is the opposite result from published observational studies. These findings require caution in their interpretation as none of the studies were powered to detect change in exacerbation rates as an outcome. Studies in usual COPD appropriately powered to detect change in exacerbation frequency often require several thousand patients.^82^ With advances in technology and a reduction in the cost of digital patient symptom diaries, there are an increasing number of studies using them to collect contemporaneous symptom data, including a recent inhaled augmentation therapy (NCT01217671) trial.^75^ This has been beneficial in usual COPD trials^83^,^84^ as it avoids recall bias and gives accurate data on severity and duration; hence, the results will be of interest. The Spanish study that focused on exacerbations had issues with missing data and also used variable treatment regimens, such that bias and lack of comparability to RCTs are an issue.^37^ There are several other reasons why the exacerbations result should be viewed with caution; first, prestudy exacerbation frequency was not reported in the three RCTs. Prior exacerbation rate predicts future exacerbation rate;^85^,^86^ hence, if a difference was present pretreatment, it could account for the observation posttreatment. Second, power calculations were not based on detecting change in exacerbation frequency. Nevertheless, this area will be important for further study because faster decline in lung function and significantly increased health care costs occur due to frequent exacerbations in usual COPD,^87^,^88^ an effect that might be more marked in AATD due to higher inflammatory burden during events.^89^

Most observational controlled studies demonstrated some differences in FEV_1_ decline with recipients of AAT augmentation benefiting over those not receiving therapy.^34^,^35^,^52^ However, such study designs have weaknesses, often relating to differences between treated and untreated groups. For instance, sex, FEV_1_, and follow-up time differences were seen between treated (German) and untreated (Dutch) patients,^34^ and age, FEV_1_, symptoms, inhaled therapies, and oxygen use differed in another study^35^ indicating that biases are likely present, which could have influenced results. In most cases, attempts were made to adjust for major differences statistically when comparing groups, and subgroup analyses suggested that benefits did not accrue in patients with FEV_1_<30% predicted. However, since it has been demonstrated that gas transfer declines more rapidly at this stage,^86^ it may simply be that different outcome measures were needed to demonstrate any effect. In one study, there was evidence that rapidly declining patients benefited more;^52^ however, there were only 11 such patients, and the definitions of slow and rapid decline utilized are not widely adopted in practice.

### COPD medical management

There was limited evidence about the efficacy of COPD treatments in AATD, and no trials reported the effects of typical treatments such as inhaled bronchodilators (long acting muscarinic antagonist, LABA, etc), steroid combinations (LABA/inhaled corticosteroids [ICS]), or PR. The available evidence was of poor quality, being prone to acquisition and potentially other biases, due to its retrospective, observational design. However, the results for influenza vaccination and self-management were at least suggestive of clinical benefit to AATD patients, thus consistent with the usual COPD literature.^22^ There is no biological reason to suppose that such interventions would differ in efficacy between usual COPD and AATD.

However, data from the uncontrolled studies^54^ and sputum work^89^ suggest that exacerbations are longer and more pro-inflammatory in AATD, such that treatments targeting their rate (eg, LABA/ICS) might be more beneficial in AATD. Recently, an unfavorable muscle response to exercise has been shown in a small number of AATD patients, compared with usual COPD,^90^ suggesting that evidence about PR might not be wholly generalizable to AATD either. This is backed up by a study of a 5-week PR program pretransplantation, where AATD patients were observed to have a smaller improvement in 6-min walking test distance compared with usual COPD (47.9 vs 60.6 m).^91^ However, the PR course was short, and the study was not eligible for inclusion in this review due to short follow-up duration; furthermore, no difference was seen after statistical adjustment for confounders. Hence, uncertainty exists about the value of COPD treatments, and further work is needed.

### COPD surgical management

AATD patients receiving LVRS demonstrated benefits, albeit inferior when compared with usual COPD. In the single study, compared to medical care (as in the National Emphysema Treatment Trial study), there were more deaths in surgically treated patients, even though their QoL improved. However, underpowering meant there were no meaningful statistics and the results should be viewed with caution. Higher mortality could reflect differences in the distribution of emphysema in AATD, making LVRS technically more difficult because access to the lung bases is needed.^92^ One small study demonstrated safety and benefits lasting up to 2 years from endobronchial valves in carefully selected patients – further investigation to reproduce these findings is warranted; however, it is encouraging and suggests that volume reduction might yet be a viable strategy. There is no evidence for endobronchial coiling in AATD yet, though suggestion of benefit in more homogeneous disease^93^ implies they could be valuable.

When compared to usual COPD, AATD lung transplantation recipients had improved survival, but this was potentially confounded by differences in age and comorbidity.^68^ Earlier onset of severe disease is well documented, and therefore, a survival benefit posttransplant may not be surprising. Two studies comparing AATD patients receiving transplant or not^71^,^72^ had conflicting results with respect to survival benefit, despite selection criteria for transplantation being similar in both countries (Sweden and UK). It is possible that more stringent matching procedures in the UK cohort^72^ explain the difference. Nevertheless, significant health status benefits occurred after transplant indicating that it is appropriate when QoL is poor. However, unlike pulmonary fibrosis where there is unified evidence of survival benefit (in part due to the poor prognosis of disease),^94^ uncertainty regarding survival benefits should be discussed with patients and is not only unique to AATD but also apparent in usual COPD and may represent the heterogeneity of disease.^94^ The single study that demonstrated decline in AATD transplant recipients included only five patients and a time frame (2 years) for the calculation of FEV_1_ decline, which is too short to be accurate; hence, the results may not be reliable.^62^ Increased posttransplant gastrointestinal complications requiring laparotomy were demonstrated in one study^63^ with those patients having a longer intensive care unit stay. There was no statistically significant effect on mortality or duration of mechanical ventilation observed, and the authors note that this was a small single-center retrospective study.

## Conclusion

There is good evidence from this systematic review that intravenous augmentation therapy slows decline in emphysema determined by CT density. This supports its use as a surrogate end point and demonstrates that augmentation remains the primary disease-specific therapy. There is paucity of data around other treatments in AATD including potential bias in the selection and reporting of clinical trials. As augmentation therapy is an expensive treatment, a full economic review is needed, and further work on optimizing patient selection for therapy could help rationalize treatment in the UK. All treatments for usual COPD may not be as efficacious in AATD due to important differences in disease process, and studies in specific treatments such as PR need to be appropriately powered to this disease group.
